# Electrocardiogram interpretation competency among emergency nurses and emergency medical service (EMS) personnel: A cross‐sectional and comparative descriptive study

**DOI:** 10.1002/nop2.809

**Published:** 2021-02-21

**Authors:** Maryam Rahimpour, Shahla Shahbazi, Mansour Ghafourifard, Neda Gilani, Cathal Breen

**Affiliations:** ^1^ Department of Medical‐Surgical Nursing Faculty of Nursing and Midwifery Tabriz University of Medical Sciences Tabriz Iran; ^2^ Department of Medical‐Surgical Nursing Faculty of Nursing and Midwifery and Clinical Research Development Unit Sina Educational, Research and Treatment Center Tabriz University of Medical Sciences Tabriz Iran; ^3^ Department of Statistics and Epidemiology Faculty of Health Tabriz University of Medical Sciences Tabriz Iran; ^4^ Institute of Nursing and Health Research School of Health Sciences Ulster University Ulster UK

**Keywords:** ECG interpretation competency, electrocardiography, emergency medical services personnel, emergency nursing, nurses

## Abstract

**Aims:**

The aim of this research study was to compare electrocardiogram (ECG) interpretation competency among emergency nurses and EMS personnel.

**Design:**

A cross‐sectional comparative descriptive study design was used.

**Methods:**

This study recruited 170 participants (105 emergency nurses and 65 EMS personnel) in northwest of Iran. Data were collected during 2018 using ECG, an interpretation competency questionnaire and analysed using SPSS V.24 through independent *t* test, linear regression, Pearson and Spearman correlation coefficient. A statistical significance of *p* < .05 was assumed.

**Results:**

The study results showed a mean score of 6.65 ± 2.16 out of 10 for emergency nurses' and 4.87 ± 1.81 for EMS personnel ECG interpretation competency (*p* < .05).

**Conclusions:**

Hospital emergency nurses were more qualified to interpret the ECG than the pre‐hospital emergency medical personnel (*p* = .792 and *β* (SE)) = 0.22 (0.84). Active involvement in ECG interpretation and standard continued education are needed to develop and improve the emergency nurses and EMS personnel ECG interpretation competency.

## 1 INTRODUCTION

Electrocardiography (ECG) is a frequent, safe and inexpensive procedure that supports arrhythmia and ischaemia diagnoses. The ECG can be indicated across a range of patient cohorts with a variety of disorders (Mobrad, 2020). Recording ECGs is essential in all hospital units, since it helps diagnose conduction and electrical heart disorders and predicts the risk of such diseases (Santana‐Santos et al., 2017). This test is also of high value in pre‐hospital emergency (Mobrad, 2020). According to Quinn et al. (2014), mortality in patients that underwent pre‐hospital ECG was lower compared with the patients without ECG monitoring across 30 days of hospitalization due to earlier detection of abnormalities and interventions to treat these (Quinn et al., 2014). As a result, considering the ever‐increasing rate of cardiovascular disease around the world (World Health Organization, 2018), it is necessary that healthcare providers are competent to record and interpret ECGs. According to the guidelines of the American College of Cardiology (ACC/AHA), an ECG should be recorded and interpreted within 10 min of referral in patients presenting with symptoms of acute coronary syndromes (ACS) (Zhang & Hsu, 2013). Moreover, improving the competency of ECG interpretation among healthcare providers who work in emergency settings is a potential patient safety issue and could minimize interpretation errors during emergency situations (Mobrad, 2020; Vand Tamadoni et al., 2020).

## 2 BACKGROUND

The competency of healthcare professionals such as doctors, nurses and emergency personnel to record and interpret ECGs to diagnose pathological disorders can assist in preventing heart disorders and decreasing the rate of mortality (Alghamdi et al., 2018; Compiet et al., 2018; Ghahramanian et al., 2020; McGrath & Sampson, 2017). Accurate ECG interpretation is an important skill for emergency nurses and EMS personnel that provide care for patients during emergency conditions ECG (Mobrad, 2020). Maintaining ECG interpretation is of great importance and a requirement of professional registration (Asghari et al., 2014; Brooks et al., 2016; Goldich, 2014; McGrath & Sampson, 2018; Quinn et al., 2014). Several studies have examined ECG interpretation competency of healthcare providers (Dulandas & Brysiewicz, 2018; Santana‐Santos et al., 2017; Stopa et al., 2017; Werner et al., 2016). Coll‐badell et al. (2017) evaluated emergency nurses' ECG interpretation competency and reported that emergency nurses demonstrated high ECG interpretation competency. Fifty‐three (93%) participants scored >7.5 points. This study reported that nurses who worked in emergency departments scored greater than 90%. Their findings did not show a statistically significant relationship between work experience in emergency unit and ECG interpretation competency, more than 50% of nurses had less than 11 years of experience in emergency departments (Coll‐Badell et al., 2017).

A study by Faramand et al. (2019) showed that EMS personnel compared with emergency nurses had lower competencies detecting ECG changes related to myocardial infarctions (MI). A review of the literature reports that despite the ubiquity of the ECG, in general healthcare professionals have inadequate ECG interpretation competency (Breen et al., 2019).

The healthcare system in Iran has recently focused on emergency units in hospitals. In recent years, the learning programme Masters of Science in Emergency Nursing has been established in some universities (Ministry of Health & Medical Education, 2019) and advanced duties such as ECG interpretation have been introduced for emergency nurses (Mirzabeygi et al., 2017). In a recent study by Hassankhani et al. (2018), emergency nurses considered the recording of 12‐lead ECG as their most frequent diagnostic role (Hassankhani et al., 2018). Upon the successful completion of a 4‐year nursing program, all students are automatically registered as “nurse” by the ministry and receive the permission work at the bedside (Shahbazi et al., 2018).

EMS in Iran has been improved in terms of its structure and work force (Shakeri et al., 2019). EMS personnel in Iran are educated through combinations of medical personnel with Associates' and B.S. degree of medical emergency, an anaesthetic technician, B.S. or M.S. degree holders of nursing, general practitioner and emergency medicine specialist (Shakeri et al., 2019). Emergency nurses and EMS personnel cooperate with each other closely (Whetzel & Wagner, 2008). As members of a medical team they act as frontline patient facing services, having competency interpreting ECG tracings is fundamental. Heart disorders have been reported as the most common reasons for admission to emergency departments and cause of mortality (Sarrafzadegan & Mohammadifard, 2019). According to the official statistics of Iran ministry of health, 33 to 39.3% of mortality rate is resulted from coronary artery diseases and it is anticipated that by 2020 this rate will have reached to 44.8% (Ministry of Health & Medical Education, 2013). Therefore, it is necessary that healthcare team personnel especially emergency nurses and EMS personnel be adequately competent in ECG acquisition and interpretation. There are a limited number of studies that compare emergency nurses' ECG interpretation competency to that of EMS personnel. In fact, in Iran, no study of ECG interpretation was found.

The aim of this study was to assess and compare the ECG interpretation competency of emergency nurses and emergency medical service (EMS) personnel working in pre‐hospital emergency centres or emergency departments of hospitals affiliated to Tabriz University of Medical Sciences, East Azerbaijan province, Iran.

## 3 THE STUDY

### 3.1 Design

This was a cross‐sectional comparative descriptive study, in which ECG interpretation competency of emergency nurses and EMS personnel was compared. This design allows the strongest evidence‐based information to answer the research question (Pilot & Beck, 2012).

## 4 METHODS

### 4.1 Sample and setting

This study was conducted across 5 hospital emergency units and 20 pre‐hospital emergency centres of Tabriz University of Medical Sciences in northwest Iran. 105 nurses employed in emergency unit and 65 EMS personnel were recruited through random sampling (Grove et al., 2012). Inclusion criteria involved: qualified with a B.S. degree in nursing, Associate's degree of medical emergency; B.S. degree in nursing or Associate's degree of Anesthesiology for EMS personnel. Numbers to recruit were determined through sample size calculation with 166 participants (105 emergency nurses and 61 EMS personnel) deemed accurate using the formula of $n_{2}=\frac{R+1}{R}.\frac{\left(S_{1}^{2}+S_{2}^{2}\right){\left(Z_{1‐\frac{\alpha }{2}}+Z_{1‐\beta }\right)}^{2}}{{\left(\bar{x_{1}}‐\bar{x_{2}}\right)}^{2}}$ (Lachin, 1981; Zar, 1984).

### 4.2 Data collection

All data for this study were collection through the completion of a two‐section questionnaire. The first section required demographic and professional variables of participants (such as age, sex, educational degree, workplace, history of clinical work in the hospital emergency unit or in pre‐hospital emergency). The second section included a validated questionnaire of ECG interpretation competency designed by Coll‐Badell et al. (2017) for measuring emergency nurses’ competency in ECG interpretation and consisted of 12 questions (Coll‐Badell et al., 2017). The permission to use that questionnaire and modify it based on the study purposes was obtained from the designers (Llaurado‐Serra M. (Personal Communication, 18 Oct 2017(. This questionnaire consisted of 2 theoretical and 10 clinical questions at various level of difficulty relating to ECG interpretation and representing the most important pathologies. The questions had multiple choices as answers. One score was assigned to each right answer, and no score was allocated to the wrong answers or to the choice of "I do not know." A maximum score of 10 could be attained with a mean of 7.5 or higher indicated acceptable ECG interpretation competency (Coll‐Badell et al., 2017).

### 4.3 Questionnaire reliability and validity

The reliability and validity of the instrument was investigated by Coll‐Badell et al. (2017), and the Cronbach's Alpha of 0.86 was reported for it (Coll‐Badell et al., 2017). In this study, firstly, the Persian version of this instrument was prepared using translation‐ back translation technique. Afterward, face and content validity was used for investigating its validity. To this end, 10 specialists including one cardiovascular specialist, one medical emergency specialist, 8 members of nursing and midwifery faculty, and one statistics specialist expressed their opinions about the questionnaire. After applying their opinions, the final Persian version was ready to use. Considering reliability, Alpha Cronbach of 0.71 was obtained for the Persian version.

### 4.4 Data collection procedures

To collect the data, the researcher (MR) attended selected teaching hospitals and pre‐hospital emergency centres during September to October 2018. After explaining the study purposes and method, participants completed informed consent forms and completed the questionnaire. To actively supervise the questionnaires completion and enhance the study accuracy, all participants were requested to complete the questionnaires in a controlled environment and return it to the researcher.

### 4.5 Data analysis

Collected data were analysed using IBM SPSS Statistics V.24. Statistical tests included independent *t* test, Pearson and Spearman correlation coefficient. P values less than 0.05 were considered to be statistical significant.

### 4.6 Ethical considerations

Before collecting the data, the Regional Research Ethics Committee of Tabriz University of Medical Sciences issued the permission for conducting this study (IR.TBZMED.REC.1397.479). The study researchers got permission for access to the study settings and conducting the data collection. Eligible participants were informed about the study purpose and procedures, data confidentiality and that they have the right to refuse to participate and could withdraw participation at any time without explanation. Participants signed informed consent forms. All data were stored securely and anonymously, and access was permitted only to the research team in accordance to institutional guidelines.

## 5 RESULTS

### 5.1 Characteristics of study participants

179 emergency nurses of 5 hospitals and EMS personnel of 20 centres were invited to participate in this study. The acceptance rate of 97.65% was attained with 170 participants completed the questionnaire. 105 participants were hospital emergency nurses, and *65* participants were EMS personnel. Participants' demographic and professional information in two groups (hospital emergency and EMS) are shown in Table 1.

**TABLE 1:** Sociodemographic and professional characteristics of the participated emergency nurses and EMS personnel (*N* = 170)

Group variables	Emergency nurses (*N* = 105)	EMS personnel (*N* = 65)
	Frequency (%)	Frequency (%)
Gender		
Male	37 (35.2)	65 (100)
Female	68 (64.8)	0 (0.0)
Education		
Associate degree (AD)	0 (0.0)	22 (33.8)
Bachelor of Science (BSc)	100 (95.2)	43 (66.2)
Master of Science (MSc)	5 (4.8)	0 (0.0)
Professional education		
Nursing	105 (100)	10 (15.4)
Emergency medical technician	0 (0.0)	46 (7.80)
Anaesthesia technicians	0 (0.0)	9 (13.8)
Type of hospital		
Referral hospital(heart specialization)	32 (18.8)	—
General Referral hospital	53 (31.2)	—
Referral hospital(non‐heart specialization)	20 (11.8)	—
Experience in coronary care unit (CCU)( Years)		
Yes	23 (21.9)	1 (1.5)
No	82 (78.1)	64 (98.5)
Has taken an ECG course?		
Yes	45 (42.9)	56 (86.2)
No	60 (57.1)	9 (13.8)
Type of training		
Face‐to‐face	45 (42.9)	56 (86.2)
No training	60 (57.1)	9 (13.8)
Considering ECG interpretation as own duty		
Yes	95 (90.5)	44 (67.7)
NO	10 (9.5)	21 (32.3)
Self‐rated ECG interpretation competency		
Weak	15(14.3)	12 (18.5)
Medium	38 (36.2)	27 (41.5)
Good	46 (43.8)	26 (40.0)
Very good	6 (5.7)	0 (0.0)
ECG interpreter		
Doctor	94 (98.5)	54 (83.1)
Nurse	1 (1.0)	5 (7.7)
Doctor or nurse	10 (9.5)	6 (9.2)
Consulting with a colleague or doctor in interpretation of ECG		
Yes	89 (84.8)	59 (90.8)
No	16 (15.2)	6 (9.2)

Abbreviations: ECG, electrocardiogram; EMS, emergency medical service.

64.8% of hospital emergency nurses were female, while 100% of EMS personnel were male. Of all participants, 21.9% of hospital emergency nurses and only 1.5% of EMS personnel had work experience in CCU. 42.9% of emergency nurses and 86.2% of EMS personnel had passed the ECG interpretation course. Of all participants, 90.5% of emergency nurses and 67.7% of EMS personnel considered ECG interpretation as their duty (Table 1).

The study results showed that hospital emergency nurses' mean (standard deviation) of ECG interpretation competency was 6.65 (2.16) and higher than that of EMS personnel, which was 4.87 (1.81). This difference was statistically significant (*p* < .05) (Table 2).

**TABLE 2:** Comparison of the competency of emergency nurses and EMS personnel in the interpretation of ECG

Statistical index group	Mean(*SD*)	Min	Max	Statistical test
Emergency nurses	6.65 (2.16)	0	10	t:5.54 *p* ^*^‐value > .001
EMS personnel	4.87 (1.81)	0.9	9.1	

Abbreviations: ECG, electrocardiogram; EMS, emergency medical service; *SD*, standard deviation.

In addition, according to our study results, only 38.1% of hospital emergency nurses and 4.6% of EMS personnel demonstrated high ECG interpretation competency and 61.9% of hospital nurses and 95.4% of EMS personnel were low competent (Table 3).

**TABLE 3:** Frequency of participants in the study based on qualification scores

Statistical index group	Competency score (<7.5)	Competency score (≤7.5)	Total
Emergency nurses	65 (61.9)	40 (38.1)	105 (100)
EMS personnel	62 (95.4)	3 (4.6)	65 (100)

Abbreviations: EMS, emergency medical service.

Furthermore, considering the threshold point of competency of 7.5, in order to compare the competency of hospital emergency nurses and EMS personnel, (after approving the qualifications of Cochran and random and independent sampling) chi‐square test was used and identical results were achieved ($\chi^{2}\left(1\right)=23.82,\;P<0.001$).

The findings of this study showed that female nurses employed in hospital emergency unit demonstrated higher ECG interpretation competency than male nurses did (*p* = .042). Additionally, the type of the hospital participants worked in was an important factor in obtaining high competency score (*p* = .001). Based on Gabriel post hoc test (considering the variance convergence and inequality of the groups' size), nurses of cardiac specialty hospitals obtained higher score compared with the general and specialty hospitals. Moreover, taking educational courses on ECG interpretation (*p* = .025), type of the course on ECG (*p* = .025) and consulting with a colleague or a doctor about ECG interpretation (*p* = .006) had a significant relationship with the competency score of hospital emergency nurses. In this study, the competency score of the nurses did not have a statistically significant relationship with their age (*p* = .825, *r* = .02) and work experience (*p* = .668, *r* = .04) based on Pearson correlation coefficient. In order to modify the effect of the variables, simultaneous modelling of the variables was utilized using General Linear Model. Accordingly, after modifying the effect of other variables, the variables of "type of the hospital" (*p* = .001), "consulting with the colleague or the doctor while interpreting" (*p* = .032) and "the rate of competency in ECG interpretation" (*p* = .018) had a significant effect on the hospital emergency nurses' competency.

The results showed that considering ECG interpretation as their duty by EMS personnel was an effective factor in obtaining high competency score (*p* = .006). Additionally, consulting with the colleague or a doctor while ECG interpretation had a statistically significant relationship with competency score of EMS personnel (*p* = .029). No statistically significant relationship was found between EMS personnel's competency score and their age (*p* =.673, *r* = .05) and work experience (*p* = .105, *r* = .20) using Pearson correlation coefficient. In order to modify the effect of the variables, simultaneous modelling of the variables was utilized using General Linear Model. Accordingly, after modifying other variables, the variables of "considering ECG interpretation as a duty" (*p* = .007) and "self‐rated ECG interpretation skill" (*p* = .021) had a significant effect on EMS personnel's score of ECG interpretation competency.

In the unadjusted linear regression model, R2 was 0.39 and adjusted R2 is 0.31. In other words, the ability to predict competence in interpreting the ECG from the variables in the model was 39%. Hospital emergency participants were more qualified to interpret the ECG than the pre‐hospital emergency medical personnel (*p* = .792 and *β* (*SE*)) = 0.22 (0.84).

Accordingly, the variables of "hospital type" (*p* =.008 and β (SE)) = 1.64(0.60), "consult with a colleague or physician in interpreting the ECG" (*p* = .022 and β (SE)) = 1.05(0.45) and "Considering ECG interpretation as own duty" (*p* = .014 and β (SE)) = 1.05(0.42) By modifying the effect of other variables, a significant effect on the competence of the participants. Hospital Emergency Participants were more qualified to interpret the ECG than prehospital emergency medical personnel (*p* = .792 and β (SE)) = 0.22(0.84).

## 6 DISCUSSION

The present study was carried out with the purpose of comparing ECG interpretation competency among hospital emergency nurses and EMS personnel. According to the available data, this study was the first study in which ECG interpretation competency of EMS personnel and hospital emergency nurses in Iran was compared. The results of the present study showed that ECG interpretation mean score in the group of hospital emergency nurses was higher than that of EMS personnel. This obvious difference indicated that hospital emergency nurses' knowledge about ECG interpretation was more than that of EMS personnel and it was probably resulted from the telemedicine systems for sending ECG from the ambulances to a doctor for interpretation (Saberian et al., 2019) that made EMS personnel unwilling to interpret ECG. This finding is shared with the findings by Faramand et al. (2019) in which EMS personnel's ECG interpretation competency was lower than that of hospital emergency nurses (Faramand et al., 2019). Similarly, the results of the study by Werner et al. (2016) showed that ambulance nurses' competency in ECG interpretation was low (Werner et al., 2016). Although according to the results of the present study, both hospital emergency nurses and EMS personnel considered ECG interpretation as their duty, both groups believed that the doctor was the main interpreter of ECG. Such uninvolvement in ECG interpretation and lack of repetition of this skill resulted in low ECG interpretation competency in healthcare professionals (Breen et al., 2019).

In this study, only 38.1% of the hospital emergency nurses and 4.6% of EMS personnel obtained the score upper 7.5 and ranked high for ECG interpretation competency. However, the findings of the study by Coll‐Badell et al. (2017) in Spain showed that the majority of emergency nurses were highly competent in ECG interpretation, as 93% of their participants obtained the score upper 7.5 using the questionnaire similar for this study (Coll‐Badell et al., 2017). Werner et al. (2016) similarly investigated EMS personnel's knowledge of ECG interpretation. The results of their study showed that almost half of the participants answered all questions correctly and only 46% of them could diagnose MI. This can have an important role in saving the patients' lives. According to the results of that study, EMS nurses had deficiencies in ECG interpretation and the only important reason of ambulance nurses' high ECG interpretation competency was their work experience in CCU (Werner et al., 2016). In both studies by Coll‐Badell et al. (2017) and Werner et al. (2016), the nurses that had worked in CCU obtained high competency score and it was in consistency with our study. EMS personnel's deficiencies and low ECG interpretation competency might affect the patients' health negatively (Coll‐Badell et al., 2017; Werner et al., 2016)..

In our study, 42.9% of hospital emergency nurses and 86.2% of EMS personnel had passed an educational course on ECG interpretation; however, competency score of EMS personnel was lower than that of hospital emergency nurses. In our study, in spite of the fact that almost more than two‐third of EMS personnel had taken part in the educational course on ECG interpretation, they were low competent in it.

In the study by Werner et al. (2016), one of the reasons why EMS personnel were low competent in ECG interpretation was that they had not participated in any educational course on ECG interpretation (Werner et al., 2016). In this regard, the results of the study by Zhang and Hsu (2013) showed that taking an educational course would increase the score of the nurses' ECG interpretation competency (Zhang & Hsu, 2013). Despite the fact that taking an educational course is helpful in improving nurses' ECG interpretation competency, not utilizing those skills might decrease their capability gradually. According to the findings of the study by Coll‐Badell et al. (2017), it is suggested that educating the nurses on ECG interpretation is repeated every 5 years (Coll‐Badell et al., 2017). Similarly, the results of the study by Morshedi (2007) in Iran showed that emergency nurses had low ECG interpretation competency compared with CCU nurses. According to this study, CCU nurses' involvement in ECG interpretation was an important factor in improving their knowledge and skill on ECG interpretation (Morshedi, 2007).‬ Recently, Alanezi (2018) in a review study with the aim of identifying the effect of educating the nurses on ECG showed that CCU nurses enjoyed higher ECG interpretation competency than other nurses did. Moreover, although, due to their experiences, CCU nurses were skilful in ECG interpretation, taking educational courses had an additional role in it (Alanezi, 2018).

In our study, type of the hospital was another factor in obtaining high ECG interpretation competency score, as the emergency nurses of cardiac specialty hospital obtained higher score than the emergency nurses of other hospitals did. Findings of Morshedi (2007) and Zhang and Hsu (2013) approved this finding (Morshedi, 2007; Zhang & Hsu, 2013). It could be because the nurses working in the emergency unit of cardiac specialty hospitals only care after the patients with heart diseases; thus involving continuously in ECG interpretation.

In contrast to our study findings, the results of the study by Stephens et al. (2007), which focused on diagnosing changes in ST segments, showed that only 20% of nurses were able to diagnose the existence of myocardial ischemia and about 19% could not diagnose MI through ST elevation. In addition, emergency nurses' ECG interpretation score was identical to that of CCU nurses. In some cases, they outperformed, though. Based on the findings of this study, continual education and interpretation of ECG could have a fundamental role in increasing nurses' ECG interpretation competency score (Stephens et al., 2007).

Results of the study by Goodridge et al. (2013) indicated a statistically significant difference in the performance of employed and contract nurses. Additionally, the results showed that nurses' needs were not met through available educational methods. Regarding the fact that all nurses are educated and have opportunity to perform this skill daily, in their study, nurses' competency was lower than expected (Goodridge et al., 2013). Findings of this study were in agreement with our study.

Despite all developments for diagnosing heart disorders, the ECG is still a valuable method and emergency nurses and EMS personnel have an indispensable position to record and interpret ECGs. According to the finding of this study, emergency nurses' and EMS personnel's low ECG interpretation competency was apparent. Both hospital emergency nurses and EMS personnel are the frontline medical teams that have contact with the patient. Any inadequacy interpreting an ECG could have a negative effect on the quality of the care for and the treatment of the patients with heart disease. To this end, arranging standard educational courses relating to ECG interpretation and providing the staff with the necessary competency before recruiting and employing them in the related units seem crucial. Considering ECG recording and interpreting as a duty and having work experience in CCU were influential factors in improving ECG interpretation. Familiarizing the personnel with their duties related to ECG recording and interpreting and utilizing the role models in CCU and/or enhancing ECG interpretation competency at first place and then recruiting the staff might be helpful in improving ECG interpretation competency. Efficiency of such steps could be investigated in further studies.

### 6.1 Study limitations

In order to actively supervise the questionnaire completion, which would result in a precise study, participants were requested to fill out the questionnaire in a controlled and a similar place and return it to the researcher. This might have made the participants answer the questions hastily. More research is required to investigate the predictors of healthcare personnel's ECG interpretation competency.

## 7 CONCLUSION

Emergency nurses and emergency medical service (EMS) personnel as frontline healthcare workers are expected to be competent to interpret an ECG. Achieving and maintaining ECG interpretation competence by healthcare professionals is recommended by the AHA/ACC as a patient safety measure. This is the one of the first studies carried out with the purpose of comparing ECG interpretation competency among hospital emergency nurses and EMS personnel. However, further research with large samples is required in Iran and other countries to validate the results of the present research. The results of the present study showed that ECG interpretation mean score of hospital emergency nurses was higher than that of EMS personnel. Considering the organizational and environmental discrepancies, educational needs of hospital nurses and EMS personnel are different and should be taken into account by the authorities. This issue is of great importance since investigating the educational needs is a primary element of planning an educational program. According to our study findings, low competency of emergency nurses and EMS personnel was noticeable. Active involvement in ECG interpretation and standard continued education are needed to develop and improve the emergency nurses and EMS personnel ECG interpretation competency. Findings of the present study might be utilized as a guide for designing the educational courses for emergency nurses and EMS personnel in order to prepare them to give care to the patients with heart disease. Since the quality of patient care in the emergency department depend on the competency of healthcare providers, an immediate development and implementation of training programs are recommended to increase the competency of EMS personnel in ECG interpretation.

## CONFLICT OF INTEREST

The authors declare that they have no known competing financial interests or personal relationships that could have appeared to influence the work reported in this paper.

## AUTHOR CONTRIBUTIONS

MR, ShSh, MGh and NG: Study design. MR: Data acquisition. NG: Analysis and interpretation of data. MR, ShSh, MGh and NG: Drafting of the article. CB and ShSh: Critical revision of the manuscript for important intellectual content. All authors contributed to final approval of the version to be submitted.

## ETHICS APPROVAL

The Regional Research Ethics Committee of Tabriz University of Medical Sciences issued the permission for conducting this study (IR.TBZMED.REC.1397.479).

## Data Availability

Data not available due to ethical restrictions.
